# Multi-Omic Approaches to Breast Cancer Metabolic Phenotyping: Applications in Diagnosis, Prognosis, and the Development of Novel Treatments

**DOI:** 10.3390/cancers13184544

**Published:** 2021-09-10

**Authors:** Nuria Gómez-Cebrián, Inés Domingo-Ortí, José Luis Poveda, María J. Vicent, Leonor Puchades-Carrasco, Antonio Pineda-Lucena

**Affiliations:** 1 Drug Discovery Unit, Instituto de Investigación Sanitaria La Fe, 46026 Valencia, Spain; nuria_gomez@iislafe.es (N.G.-C.); idomingo@cipf.es (I.D.-O.); 2 Polymer Therapeutic Laboratory, Centro de Investigación Príncipe Felipe, 46012 Valencia, Spain; mjvicent@cipf.es; 3 Pharmacy Department, Hospital Universitario y Politécnico La Fe, 46026 Valencia, Spain; poveda_josand@gva.es; 4 Molecular Therapeutics Program, Centro de Investigación Médica Aplicada, 31008 Navarra, Spain

**Keywords:** metabolism, breast cancer, multi-omics, biomarkers, early diagnosis, subtyping, prognosis, treatment

## Abstract

**Simple Summary:**

Breast cancer (BC) is a heterogeneous tumor type and has become the leading cause of cancer worldwide, with 685,000 deaths forecast in 2020. The clinical management of BC patients remains challenging, and there exists an urgent need for improved diagnostic, prognostic, and therapeutic strategies. Multi-omics platforms represent a promising tool for discovering novel biomarkers and identifying new therapeutic targets. In addition, the ongoing development of multi-omics approaches may foster the identification of more robust and accurate algorithms for data analysis. This review aims to summarize the results of recent multi-omics-based studies focused on the characterization of the metabolic phenotype of BC.

**Abstract:**

Breast cancer (BC) is characterized by high disease heterogeneity and represents the most frequently diagnosed cancer among women worldwide. Complex and subtype-specific gene expression alterations participate in disease development and progression, with BC cells known to rewire their cellular metabolism to survive, proliferate, and invade. Hence, as an emerging cancer hallmark, metabolic reprogramming holds great promise for cancer diagnosis, prognosis, and treatment. Multi-omics approaches (the combined analysis of various types of omics data) offer opportunities to advance our understanding of the molecular changes underlying metabolic rewiring in complex diseases such as BC. Recent studies focusing on the combined analysis of genomics, epigenomics, transcriptomics, proteomics, and/or metabolomics in different BC subtypes have provided novel insights into the specificities of metabolic rewiring and the vulnerabilities that may guide therapeutic development and improve patient outcomes. This review summarizes the findings of multi-omics studies focused on the characterization of the specific metabolic phenotypes of BC and discusses how they may improve clinical BC diagnosis, subtyping, and treatment.

## 1. Introduction

Breast cancer (BC) is the most frequently diagnosed tumor and the leading cause of cancer deaths in women worldwide [1]. The year 2020 saw an estimated 2.3 million new cases of BC (11.7% of all cancer cases), with 685,000 deaths worldwide [1]; however, advances in population screening and early treatment (among other factors) have supported a steady decrease in BC mortality [2,3]. Unfortunately, figures from the American Cancer Society place the five-year survival rate after diagnosis of metastatic BC at 27%, a low value considering the 99% five-year survival rate for localized disease [4]. Therefore, early BC detection represents a crucial step in reducing disease mortality [5]. BC screening currently relies on mammography, a non-invasive strategy primarily performed in women between 50 to 69 years of age that has prompted a reduction in BC mortality [6]. Nevertheless, this approach suffers from several limitations, including false-positive reporting and overdiagnosis [6,7,8,9,10]. Ultrasound, magnetic resonance imaging, and computed tomography can overcome such problems thanks to their high sensitivity; however, the elevated costs associated with these tools make this approach less accessible. Thus, we still lack alternative methods for the accurate, non-invasive, and low-cost diagnosis of early-stage BC.

BC is a highly heterogeneous disease from a molecular perspective and is primarily characterized by the overexpression of the HER2 growth factor, estrogen receptor (ER), and progesterone receptor (PR) and mutations in the *BRCA1/2* genes, with the latter associated with a higher risk of developing BC [11]. Former classifications of BC tumors employed tumor size, histological grade, immunohistochemistry of ER/PR status, and the amplification of HER2. The addition of gene expression profiling to these molecular features has resulted in the classification currently used by the European Society for Medical Oncology as a clinical guideline for BC diagnosis, follow-up, and treatment [12,13]. This system classifies BC tumors into four major intrinsic molecular subgroups: luminal A (ER+ and/or PR+, HER2-, low Ki67), luminal B (ER+ and/or PR+, HER2+ or HER2- with high Ki67), basal-like (ER/PR-, HER2-), and HER2-enriched (ER/PR-, HER2+). Luminal A tumors (the low-grade group) are the most common BC subtype, comprising over 60–70% of all cases. Meanwhile, basal-like tumors, with an ~80% overlap with highly proliferative triple-negative breast cancer (TNBC) [14], exhibit aggressive behavior and suffer from poor prognosis [15]. Although ER+ tumors present lower recurrence rates within the first five years, over 50% of tumor recurrences occur after this time and cause most BC-related deaths [16,17]. Each BC subtype has a characteristic biological profile, prognosis, and treatment strategy [18,19,20,21,22], and several scoring systems aid prognosis and treatment decision-making processes. Unfortunately, systems based on different molecular features related to tumor biology, including histological type, grade, lymphovascular invasion, and marker status, do not accurately reflect BC subtype heterogeneity or specific patient subtypes [23]. Thus, enormous efforts have been devoted to classifying heterogeneous BC subtypes into molecular subtypes that guide treatment decisions [24,25,26,27,28].

Current BC treatment strategies are applied according to BC subtype and prognosis. BC subtypes expressing hormone receptors (Luminal A, Luminal B, and HER2+) associate with good/intermediate prognosis, and their clinical management includes endocrine therapy alone or in combination with chemotherapy if there exists the risk of recurrence. Non-metastatic BC treatment also includes surgical resection and postoperative radiation therapy. Nevertheless, we lack effective therapeutic options for TNBC, the most aggressive BC subtype [29,30,31], and we urgently require the development of novel treatment strategies specifically targeting TNBC tumors.

Importantly, a range of studies employing multi-omics-based approaches, including genomics, epigenomics, transcriptomics, proteomics, and metabolomics, have noted the existence of characteristic metabolic profiles for BC subtypes. Metabolomics-based studies performed in BC preclinical models [32,33] and patient tissues [34,35] have identified alterations to metabolites associated with glutamine, glutathione, and choline (Cho) metabolism. Similar studies have been carried out in biofluids such as urine [36,37], blood [38,39], and saliva [40], while other studies focusing on the metabolic characterization of normal breast epithelial and BC cell lines have revealed specific metabolomic differences between TNBC, Luminal B, and HER2+ subtypes [41].

Alterations to metabolic pathways associated with BC tumors and disease progression have been broadly explored at the genomic level [42,43,44]. Transcriptomic-based approaches have characterized specific metabolic changes in different BC subtypes [41] and evaluated the impact of various treatments on metabolic processes [45]. Furthermore, proteomics studies have revealed alterations in metabolism-associated protein expression in BC patients and correlations to overall and recurrence-free survival [46].

**Figure 1:**
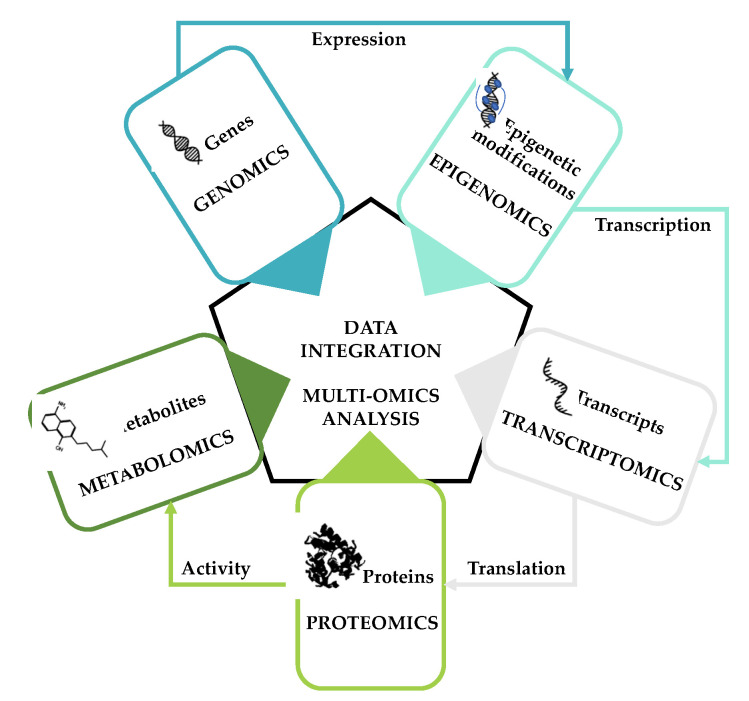
Schematic representation of the most commonly used omic platforms for multi-omics studies.

The combination of omic approaches has recently emerged as a promising strategy for generating information on the interrelationship between genetic aberrations, epigenetic alterations, changes in gene transcription and signaling pathways, and metabolic alterations that may contribute to the progression of a specific condition (Figure 1).

Interestingly, a range of studies has applied multi-omics approaches to the study of BC metabolism and has provided a more accurate understanding of the disease and its sub-classification [47,48,49]. These studies enabled the identification of characteristic disease biomarkers [50] or metabolic pathways related to specific subgroups of BC patients [51]. This review summarizes the results obtained in recently published multi-omics-based studies focused on analyzing those metabolic changes related to BC progression.

## 2. BC Diagnosis

Potential metabolic biomarkers for the detection and stratification of BC patients have been described in genomic [42], epigenomic [52,53], transcriptomic [54], proteomic [55], and metabolomic [34,35,36,37,38,39,40,41,56,57,58] studies. However, multi-omics-based analyses have been carried out in a reduced number of studies, which probably reflects limited sample availability and/or technical difficulties associated with generating complete multi-omics datasets. Thirteen studies published between 2010 and 2021 have integrated two different omics datasets to identify disease-associated metabolic alterations that could improve the diagnosis and subclassification of BC patients [48,57,59,60,61,62,63,64,65,66,67,68,69]. Tissue samples represented the most frequently used sample type, with only three studies collecting biofluids (primarily blood). Most cases evaluated a combination of transcriptomics and metabolomics, while one study included lipidomics, a recently emerged discipline in the omics field [70].

### 2.1. BC Metabolic Markers

The identification of novel biomarkers useful for early detection remains a critical clinical challenge in BC. A range of studies has compared the metabolic profiles of BC patients and healthy individuals using multi-omics approaches to identify potential metabolic markers with clinical utility in early BC diagnosis (Table 1). Integration of transcriptomics and metabolomics datasets represent the primary strategy followed in these studies, with tissue samples the preferable choice for analysis. Overall, the characterization of metabolic differences between BC and healthy tissues has revealed alterations in metabolites and/or enzymes involved in glycolysis, amino acid, lipid, and nucleotide metabolism.

A multi-omics study conducted by Iqbal et al. described the role of chromobox (CBX) proteins in BC metabolic reprogramming [59]. The authors identified a significant correlation between glycolytic activity and changes in *CBX2* and *CBX7* isoforms levels. Glycolytic metabolites, including glucose-6-phosphate, 3-phosphoglycerate, and fructose-6-phosphate, exhibited upregulated expression in high *CBX2* and low *CBX7* tumor samples. A study focused on the characterization of specific dysregulations in lipid metabolism conducted by Hilvo et al. reported increased levels of phospholipids, sphingomyelins, and ceramides, and the overexpression of four enzymes involved in de novo lipid metabolism (acetyl-CoA carboxylase alpha (*ACACA*)*,* fatty acid synthase (*FASN*)*,* insulin-induced gene 1 (*INSIG1*), and sterol regulatory element-binding transcription factor 1 (*SREBP1*)) in BC compared to healthy tissues [60]. Enhanced phospholipid metabolism in BC tissues has been previously observed, reflected by high phosphatidylcholine (PtdCho), phosphatidylethanolamine, or phosphocholine (PCho) levels [71,72,73]. In addition, reports suggest that certain of the above-mentioned lipid metabolism enzymes play a significant role in BC progression. For instance, acetyl-CoA carboxylase alpha (*ACACA*) overexpression associates with increased cancer risk [74] and can be detected in early-stage BC [75], while fatty acid synthase (*FASN*) promoted BC metastasis [76,77] and was proposed as a potential therapeutic target for BC treatment [78].

Additional multi-omics studies have focused on analyzing metabolic alterations in plasma samples from BC patients and correlating them with transcriptomic changes in BC tumors. Luo and coworkers identified significant changes in purine metabolism when combining data from transcriptomics and metabolomics analyses of BC [61]. Enrichment-pathway analysis demonstrated the lower expression of genes related to purine nucleotides degradation. Notably, the authors also reported the downregulation of purine nucleoside phosphorylase (*PNP*) and hypoxanthine phosphoribosyltransferase (*HPRT1*) expression, two enzymes involved in the purine salvage pathway, as alterations in this pathway. Furthermore, the authors noted lower guanine and hypoxanthine levels in BC tissues. Although studies have suggested that de novo nucleotide synthesis can promote BC metastasis [79], recent studies have reported the downregulation of genes involved in salvage synthesis concurrent with the upregulated expression of nucleotide de novo synthesis genes in metastatic BC cells [79]. Related studies suggested the overexpression of ecto-5’-nucleotidase (*CD73*, which catalyzes the dephosphorylation of adenosine monophosphates into adenosine) in BC cells and an associated modulation of tumor growth and metastasis [80,81]; meanwhile, the observed increased activity of adenosine deaminase (ADA) in BC tumors [82,83,84] may represent a diagnostic tool for BC [83,84]. Additionally, enzymes involved in purine metabolism have also been proposed as potential drug targets for BC, and these include the ribonucleotide reductase regulatory subunit M2 (*RRM2*), natriuretic peptide receptor 1 (*NPR1*,) and phosphodiesterases (*PDE*) enzymes [54,85].

Multi-omics approaches have also been explored in the development of novel biomarker combinations that discriminate healthy tissue from BC at different stages [62]. In a multi-omics study aiming to discover crucial metabolic pathway signatures for more accurate BC diagnosis, Huang et al. reported an eight pathway-based classification model that provided highly accurate predictions in all-stage and early-stage BC patients [57]. Taurine and hypotaurine metabolism and the alanine, aspartate, and glutamate pathway were identified as critical to the early diagnosis of BC, with consistent changes occurring in plasma and serum metabolomics analyses and the transcriptomics-based analyses of BC tumors and adjacent normal tissue. The study also reported significant correlations between alterations to metabolite concentrations and gene expression levels when combining both omics approaches. For instance, increased asparagine, cysteine, and hypotaurine concentrations in early-stage BC correlated with the overexpression of the relevant synthetic enzymes, while lower levels of oxoglutarate associated with a significant decrease in the expression of the enzymes involved in its generation. Recent metabolomics studies have also reported altered taurine levels in plasma and serum samples of BC patients [86] and altered asparagine and cysteine levels in blood [87,88] and urine [36] of BC patients.

Dowling et al. evaluated protein and metabolite levels in BC serum samples using high-throughput antibody-based screening and mass spectrometry platforms, respectively [62]. A combined analysis demonstrated that the addition of glutamate, 12-hydroxyeicosatetraenoic acid, β-hydroxybutyrate, coagulation factor V, and matrix metalloproteinase-1 to cancer antigen 15-3 (CA15-3) values, a widely used serum marker in BC, permitted accurate discrimination when comparing non-malignant breast disease and BC at different stages. This combination resulted in noticeably improved prediction values compared with a classification based only on CA15-3 alone, confirming the potential of multi-omics approaches to provide clinically relevant information.

### 2.2. BC Metabolic Subtyping

Multi-omics approaches have also shown great potential for redefining cancer subtypes [89,90]. Recent studies have focused on exploiting the information derived from multi-omics strategies to improve tumor subtyping (Table 2). Most of these studies, which relied on tissue sample analysis and the integration of transcriptomics and metabolomics data, focused on the characterization of ER- vs. ER+ BC tumors and basal- vs. luminal-like BC tumors, while a few cases explored unsupervised BC subtyping. Overall, metabolic alterations in glycolysis, the tricarboxylic acid (TCA) cycle, the pentose phosphate pathway (PPP), and lipid, nucleotide, and amino acid metabolism distinguished different BC tumor subtypes.

The results of different multi-omics studies reveal common alterations to lipid, nucleotide, and amino acid metabolism when comparing basal- and luminal-like BC subtypes, and Moestue and coworkers focused on differences in Cho metabolism profiles between these two BC subtypes [63]. In this study, basal-like BC models displayed higher concentrations of glycerophosphocholine (GPC) than PCho and higher glycine concentrations, which could be explained by lower choline kinase (*CHKA*, *CHKB*) expression and higher PtdCho degradation mediated by the overexpression of phospholipase A2 group 4A (*PLA2G4A*) and phospholipase B1 (*PLB1*). This group of tumors also exhibited a characteristic metabolic shift from PtdCho synthesis to glycine formation mediated by choline dehydrogenase (*CHDH*) and sarcosine dehydrogenase (*SARDH*) expression. A related metabolomics study established increased GPC and decreased PCho concentration levels in TNBC samples compared with luminal A subtypes [35]. In addition, BC patients exhibiting better prognoses exhibited lower concentrations of glycine than those with worse prognoses [91]. In another study, Grinde and colleagues discovered that the regulation of Cho metabolism varied between different BC molecular subgroups [64]. The authors analyzed the metabolomic and transcriptomic profiles of thirty-four patient-derived xenograft BC models to explore the differences between basal- and luminal-like subgroups and revealed a higher PCho/GPC ratio in luminal B tumors. The Cho metabolic profiles and the expression of genes involved in Cho metabolism, including *CHKA* and glycerophosphodiester phosphodiesterase domain containing 5 (*GDPD5*), displayed differences in luminal B and basal-like xenografts. Overall, the results of these and other studies highlight the heterogeneity in Cho metabolism in different BC molecular subtypes [92,93].

The luminal- and basal-like BC subgroups also display additional characteristic metabolic differences. Putluri et al. used an integrated analysis of the metabolome and transcriptome of these subgroups to identify eleven differentially expressed metabolic pathways between the luminal- and basal-like BC subgroups, including nucleotide (purine and pyrimidine) synthesis and different amino acid metabolism pathways, such as lysine degradation or branched-chain amino acid (BCAA) and glutamate metabolism [65]. The increased glutamate and nucleotide metabolism observed in basal-like tumors agree with previous studies describing a higher susceptibility to glutaminolysis-targeting therapies in TNBC [94,95] and an enhanced de novo nucleotide synthesis in metastatic BC [79]. Enhanced pyrimidine metabolism in TNBC has been reported [35], and inhibition of this metabolic route represents a potential therapeutic strategy to promote the sensitivity of TNBC cells to chemotherapy by exacerbating DNA damage [96].

Metabolic differences between basal and luminal-like BC phenotypes were recently explored by Mahendralingam and colleagues in a study based on a combined proteomic and transcriptomic analysis of normal mammary epithelial cells (MECs) [66]. The authors identified distinct metabolic phenotypes corresponding to basal, luminal progenitor, and mature luminal cells. Specifically, basal-like cells exhibited a glycolytic phenotype characterized by the abundance of glycolytic enzymes (phosphofructokinase (muscle) (*PFKM*)*,* aldolase, fructose-bisphosphate C (*ALDOC*)*,* glyceraldehyde-3-phosphate dehydrogenase (*GAPDH*), and pyruvate kinase M1/2 (*PKM*)), while luminal progenitors exhibited enhanced oxidative phosphorylation (OXPHOS). Bioinformatics analyses indicated that BC subtypes retain metabolic characteristics of their cell of origin. These findings agree with recent multi-omics studies that observed increased OXPHOS activity in TNBC, whose cells of origin are luminal progenitors [69].

Multiple studies have revealed relevant metabolic differences between the ER- and ER+ BC subtypes using multi-omics approaches. Using a combination of genomics, transcriptomics, and metabolomics, Tang and coworkers reported correlations between gene mutations/gene expression levels and tumor metabolism [67]. Cancers with TP53 alterations exhibited decreased levels of lipid glycerophosphocholines, tumors with ERBB2 amplification associated with changes in docosapentaenoate, fucose, and 1-oleoylglycerophosphoethanolamine levels, and *PIK3CA* mutations correlated with altered malonylcarnitine levels. Among other significant associations, indoleamine 2,3-dioxygenase 1 (*IDO1*) overexpression correlated with increased kynurenine levels. *IDO1* functions by generating kynurenine from tryptophan, and a higher kynurenine/tryptophan ratio was observed during the progression of tumors, including BC [97,98]. Overall, Tang et al. established an increase in eight metabolites in ER+ tumors (including carnitine derivates and short- and medium-chain fatty acids) and an increase in long-chain fatty acid and monoacylglycerol levels in ER- tumors, indicating that differences in lipolysis and fatty acid oxidation (FAO) correlate with hormone receptor status [67]. Furthermore, higher *BRCA1* expression levels associated with changes in a set of metabolites reflecting FAO activation in these tumors. FAO-related genes appear to promote cell proliferation and survival [99,100], and FAO inhibition has been proposed as a potential therapeutic strategy for BC treatment [101,102,103].

In a more recent study, Barupal et al. used reactome pathway mapping analyses to associate alterations to glycolysis, PPP, TCA cycle, nucleotide salvage, glutathione conjugation, steroid metabolism, fatty acyl-CoA biosynthesis, serine biosynthesis, and metabolism of aromatic amino acids with the ER- phenotype [68]. Increased PPP activity represented the most significant change supported by the multi-omics analysis. The PPP intermediates ribose-5-phosphate and ribulose-5-phosphate increased in ER- tumors and associated with increased protein and mRNA expression for the critical oxidation enzymes glucose-6-phosphate dehydrogenase (*G6PD*) and 6-phosphogluconate dehydrogenase (*PGD*). Furthermore, ER- tumors also displayed increased levels of transketolase (*TKT*), a key enzyme in the non-oxidative branch of the PPP, phosphoglucomutase 1 (*PGM1*), ribose-5-phosphate isomerase (*RPIA*), and deoxyribose-phosphate aldolase (*DERA*). The PPP delivers the nicotinamide adenine nucleotide phosphate (NADPH) and pentose phosphates required for nucleotide and fatty acid synthesis during cell division and tumor proliferation [104,105]. Consistent with PPP activation, the study discovered elevated levels of five purine metabolites (adenine, guanosine, guanine, xanthine, and hypoxanthine). In addition, β-alanine (an intermediate of the pyrimidine salvage pathway) and uracil, pseudo-uridine, uridine monophosphate (UMP), and cytidine monophosphate (CMP) levels also displayed increased expression in ER- tumors [68]. Interestingly, Budczies et al. previously described β-alanine as a significant marker differentiating ER- and ER+ BC [106]. Additional studies reported significant increases in lipid content, including phospholipids, in ER- compared with ER+ tumors [60,107]. Based on the analysis of lipidomics and gene expression data from 257 human BC tissues, Hilvo et al. associated alterations in phospholipid metabolism with ER status and tumor grade [60]. In this study, both ER- and high-grade tumors exhibited increased levels of phospholipids that incorporated de novo synthesized palmitate and myristic acid, while only tumor grade (and not ER- status) associated with changes in lipid-related gene expression.

Related studies employed integrated approaches to metabolically identify potential BC subgroups following an unsupervised approach. Haukaas et al. applied hierarchical clustering on 228 primary BC samples and identified three different metabolic subgroups (MC1, MC2, and MC3) [48]. Integrated pathway analysis of metabolite and gene expression data uncovered differences in glycolysis/gluconeogenesis and glycerophospholipid metabolism between the clusters. MC1 exhibited a lipidomic phenotype with higher levels of GPC and PCho; MC2 displayed a low glycolytic profile exhibiting increased glucose concentrations; and MC3 exhibited elevated lactate and alanine levels. Compared with the other two subgroups, MC1 possessed increased levels of GPC and PCho due to the downregulation of genes involved in PtdCho degradation and higher *CHKA* levels. In addition, the MC1 cluster exhibited lower acetate and glutamine levels due to the lower expression levels of aldehyde dehydrogenase *(ALDH)* and glutaminase (*GLS)* enzymes, respectively. However, the study failed to encounter differences in mRNA levels between MC2 and MC3 subgroups.

More recently, Gong et al. applied a similar approach to identify metabolic pathway-based subtypes (MPSs) in TNBC and identified three different metabolic phenotypes (lipogenic, glycolytic, and mixed subtypes) with distinct molecular features and sensitivities to various metabolic inhibitors [69]. In particular, the lipogenic phenotype (MPS1) associated with the high expression of genes involved in cholesterol and de novo lipid metabolism and the accumulation of several lipids, while the glycolytic group (MPS2) exhibited the upregulation of genes involved in glycolysis and increased concentrations of metabolic intermediates of glycolysis and nucleotide metabolism. The authors also evaluated the mRNA expression of metabolic enzymes and corresponding metabolite abundance to assess their impact on the MPS classification. Tumors corresponding to the MPS1 subtype displayed increased expression of acetyl-CoA carboxylase alpha (*ACACA*), HMG-CoA reductase (*HMGCR*), *FASN*, and stearoyl-CoA desaturase (*SCD*), and a significant accumulation of various lipids, such as myristic, palmitoleic, oleic, and arachidonic acid. In contrast, the MSP2 subtype displayed the elevated expression of metabolic enzymes associated with glycolysis and nucleotide metabolism, with the most significant changes associated with phosphofructokinase (*PFKP*), enolase 2 (*ENO2*), thymidylate synthetase (*TYMS*), CTP synthase 1 (*CTPS1*), but also with glucose transporter (*GLUD*), solute carrier family 2 member 1 (*SLC2A1*), and lactate transporter *SLC16A1*. The MSP2 subtype also displayed lower levels of glucose and the accumulation of intermediates in glycolysis and nucleotide metabolism, including glucose-1-phosphate, dihydroxyacetone phosphate, lactate, and adenosine-3′-5′-diphosphate. MPS3, the mixed subtype, showed only partial pathway dysregulation.

## 3. Multi-Omics Studies of BC Prognosis

**Figure 2:**
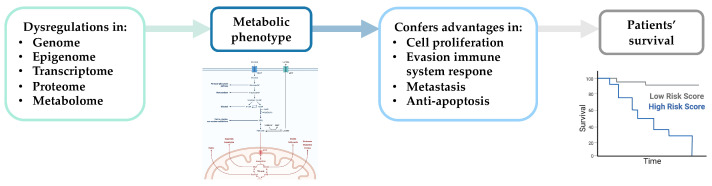
Schematic representation of the impact of metabolic changes on essential molecular processes associated with tumor progression and patient survival. Created with BioRender.com.

Metabolic deregulation can impact various molecular processes (e.g., cell proliferation, apoptosis, migration, and invasion) that contribute to tumor progression [44,108,109,110] and influence cancer patient survival (Figure 2) [111].

Several studies have reported associations between metabolic alterations and BC patient survival based on single omic analysis, including genomic [112], transcriptomic [113], proteomic [46], and metabolomic [56,114,115] studies in tissue [46,112,113] and serum [56,114,115] samples. However, additional studies based on the integration of data from multi-omics analyses have provided more accurate information regarding the molecules involved in metabolic rewiring associated with BC progression. Of the seven studies following a multi-omics approach to identify metabolic alterations associated with BC prognosis (Table 3), most relied on the analysis of tissue sample analysis and the integration of transcriptomic and metabolomic datasets. Overall, BC patient survival associated with the altered expression of enzymes involved in nucleotide, lipid, and amino acid metabolism.

Previous studies have demonstrated that nucleotide biosynthesis plays a vital role in BC [79,80,83,84,117], and could represent a promising therapeutic strategy [54,85,118]. Notably, two of the multi-omics studies included in Table 3 established an inverse correlation between the expression levels of genes involved in de novo purine and pyrimidine syntheses and BC patient survival [61,65]. Putluri et al. performed an in silico analysis to evaluate the association between omics-based enrichment and patient survival, using ten independent gene expression data sets to select clinically relevant prognostic biomarkers [65]. Kaplan–Meyer curves revealed an association between increased expression of pyrimidine metabolism-related genes and shorter metastasis-free survival across all BC and within the subset of ER + tumors. *RRM2*, a critical gene in pyrimidine metabolism displaying elevated expression in aggressive BC [119], has prognostic relevance in BC [120,121] when combined with proliferation markers [122]. *RRM2* expression distinguished good vs. poor survival within the entire BC patient group in this multi-omics study, including a significant proportion of luminal A subtype typically considered to have better survival outcomes [80]. Luo and colleagues integrated metabolomic and transcriptomic analysis to confirm the association between alterations in nucleotide metabolism and BC patient survival in a TCGA cohort of patients [61]. The authors observed a significant correlation between poor survival of BC patients and changes to the expression of *RRM2* and adenosine monophosphate deaminase 1 (*AMPD1*), a key enzyme in de novo purine synthesis. These enzymes have been postulated as promising therapeutic targets in different tumor types, including BC [65,85,123,124,125,126].

Iqbal et al. established antagonistic roles of CBX2 and CBX7 in metabolic reprogramming of BC and an association with BC patient survival [59]. The authors described a significant correlation between higher *CBX2* and lower *CBX7* mRNA levels and worse BC prognosis, which agrees with previous findings that correlated the *CBX2* or *CBX7* expression with overall patient survival [127,128,129,130].

As for the alterations in lipid metabolism, Camarda and colleagues followed a targeted metabolomics approach and reported the dramatic upregulation of FAO intermediates in a MYC-driven model of TNBC [102]. To characterize a potential association between FAO gene expression and prognosis in TNBC, the authors performed a univariate analysis of 336 fatty acid metabolism genes on a patient cohort with long-term distant recurrence-free survival data. The analysis revealed that decreased *ACACB* (acetyl-CoA carboxylase 2, ACC2) expression levels associated with worse prognoses in all BC and TNBC patients. Subsequent studies also described significant associations between increased levels of ACC2 and better BC prognosis [131,132,133,134]. Kang et al. conducted a multi-layered lipidomics and transcriptomics analysis to describe the rewiring of the BC lipidome during malignant transformation [116]. Analyses in a spheroid-induced epithelial-mesenchymal transition (EMT) model demonstrated a dramatic reduction in the ratio of C22:6n3 (docosahexaenoic acid, DHA) to C22:5n3 in spheroid cells, similarly to the down-regulation of *ELOVL2*, a process associated with the induction of metastatic characteristics in BC cells. The authors examined the relationship between *ELOVL2* expression and metastatic relapse-free in a BC cohort with a follow-up of ten years, resulting in the discovery of an association between lower *ELOVL2* expression levels and shorter metastasis-free survival and higher tumor grade [116]. A recent study investigating the molecular mechanisms of tamoxifen resistance in BC confirmed these findings and described lower *ELOVL2* expression in tamoxifen-resistant models and *ELOVL2* downregulation in patients with tamoxifen resistance [135].

Additional studies have revealed a correlation between alterations in the levels of genes and metabolites involved in amino acid metabolism and BC prognosis. Terunuma and coworkers identified a subset of BC tumors accumulating high levels of 2-hydroxyglutarate (2HG). Further analyses revealed the presence of a subgroup of BC patients with significantly decreased survival characterized by an exceptionally high accumulation of 2HG, reduced DNA methylation at the isocitrate dehydrogenase (*IDH2)* locus, increased *IDH2* expression, and increased levels of S-adenosyl- methionine (SAM) and S-adenosylhomocysteine (SAH) [107]. Previous studies reported an accumulation of the oncometabolite 2HG in different tumor types, including BC [67], glioma [136], and leukemia [137]. In another study, concerning amino acid metabolism, Budczies et al. described an association between alterations in the metabolism of β-alanine and shorter recurrence-free survival of BC patients [106]. Specifically, the authors demonstrated that lower expression levels of 4-aminobutyrate aminotransferase (*ABAT*), which negatively correlated with the concentration of β-alanine, indicated worse prognoses in BC patients. A similar study reported decreased *ABAT* expression in more aggressive BC subtypes, which correlated with an increased risk of metastasis and shorter overall, relapse-free, and distant metastasis-free survival [138]. Finally, Jansen et al. correlated *ABAT* downregulation with poor progression-free and metastasis-free survival in tamoxifen-treated patients [139].

## 4. Multi-Omics Studies and Novel BC Treatment Strategies

**Figure 3:**
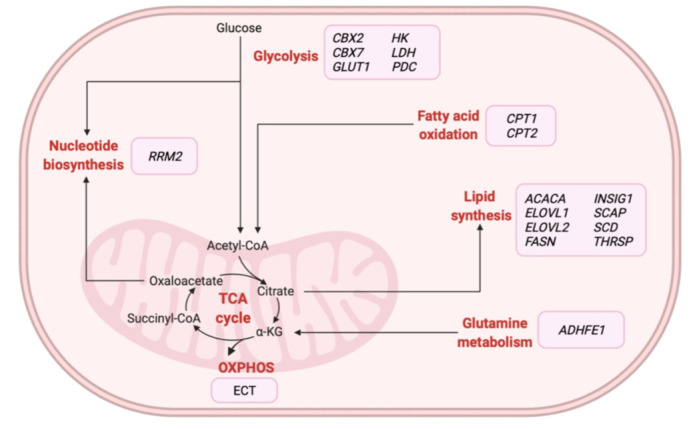
Overview of metabolic-related therapeutic targets for the treatment of BC patients identified from multi-omics-based studies. α-KG: alpha-ketoglutarate, *ACACA*: acetyl-CoA carboxylase alpha, *ADHFE1*: alcohol dehydrogenase iron containing 1, *CBX2*: chromobox 2, *CBX7*: chromobox 7, *CPT1*: carnitine palmitoyltransferase 1A, *CPT2*: carnitine palmitoyltransferase 2, ECT: electron transport chain, *ELOVL1*: ELOVL fatty acid elongase 1, *ELOVL2*: ELOVL fatty acid elongase 2, *FASN*: fatty acid synthase, *GLUT1*: glucose transporter 1, *HK*: hexokinase, *INSIG1*: insulin-induced gene 1, *LDH*: lactate dehydrogenase, OXPHOS: oxidative phosphorylation, *PDC*: pyruvate dehydrogenase kinase, *RRM2*: ribonucleotide reductase regulatory subunit M2, *SCAP*: SREBF chaperone, *SCD*: stearoyl-CoA desaturase, TCA: tricarboxylic acid, *THRSP*: thyroid hormone-responsive. Created with BioRender.com.

Omics-based technologies have also been used to identify novel therapeutic targets and monitor biological alterations related to BC metabolism following treatment [32,45,140,141,142,143,144,145,146]. The majority of studies relied on the application of metabolomics-based approaches in BC tissue [141,143] and serum [140,142,144] samples, although groups have evaluated transcriptomic [33,45] and proteomic [146] profiles in tissue samples. Various studies have described how the combination of omics approaches could characterize specific targets and foster the development of novel therapeutic strategies for specific subgroups of BC patients [147,148,149]. In particular, multi-omics studies have focused on identifying and validating metabolic enzymes as promising therapeutic strategies for the treatment of different BC tumors (Table 4). Figure 3 illustrates those metabolic-related genes proposed as potential therapeutic targets for treating BC patients in these studies. Overall, these findings suggest the therapeutic potential of inhibiting specific metabolic enzymes associated with glycolysis or involved in nucleotide, amino acid, and lipid metabolism in BC patients.

Iqbal and coworkers demonstrated that silencing *CBX2* and *CBX7* exhibited inverse effects on glycolysis, ATP production, viability, and proliferation [59]. *CBX7* overexpression provided comparable results to *CBX2* knockdown, which included decreased biomass production and reduced cell viability and proliferation. These in vitro results agreed with the findings of the transcripto-metabolomic analyses performed on BC patients and validated the roles of *CBX2* and *CBX7* in metabolic reprogramming of BC, highlighting the potential of these targets for the development of therapeutic strategies in BC. Of note, additional studies have provided similar results for CBX2 and CBX7 in BC [130,150,151], pancreatic adenocarcinoma [128] and metastatic prostate cancer [152]. Gong and coworkers evaluated the sensitivity of different BC metabolic phenotypes to metabolic inhibitors targeting glycolysis or de novo fatty acid synthesis [69]. The glycolytic BC phenotype displayed greater sensitivity to glycolytic inhibitors (oxamate, lactate dehydrogenase (*LDH*) inhibitors, and 2-deoxy-D-glucose), while inhibitors of lipid synthesis (cerulin and *FASN* inhibitor) exhibited higher efficacy against the lipogenic phenotype. Significantly, in vivo *LDH* inhibition enhanced tumor response to anti-PD-1 immunotherapy in the BC glycolytic phenotype. Previous studies have shown that *LDH* inhibition can suppress glycolysis [153,154] and cell proliferation [155] in BC cell lines. Differences in sensitivity to pharmacological inhibitors targeting glycolysis or electron transport chain (ECT) subunits were evaluated in different metabolic MECs phenotypes by Mahendralingam and colleagues [66]. In this study, the glycolytic phenotype displayed greater sensitivity to inhibitors targeting glucose transporter 1 (*GLUT1*), hexokinase (*HK*), *LDH,* and pyruvate dehydrogenase kinase (*PDC*), which agrees with results reported by Gong et al. [69]. Furthermore, studies have demonstrated that *HK* inhibition prevents BC growth [156,157].

The metabolic enzyme *RRM2* has also been proposed as a potential therapeutic target for the treatment of BC. Putluri et al. observed that inhibiting *RRM2* in BC cells significantly decreased proliferation and the expression of cell cycle genes and sensitized cells to tamoxifen treatment [65]. In agreement with the potential relevance of *RRM2*, additional studies have reported a reduction in proliferation [85,126] and tamoxifen resistance [158] in BC cell lines following *RRM2* inhibition. Furthermore, associations between *RRM2* overexpression and deterioration in BC survival have been widely reported, strongly suggesting a role as a targeted therapy for BC [61,120,121].

A multi-omics-based study by Terunuma et al. identified alterations associated with glutamine metabolism in a subset of BC tumors [107]. The authors described a subset of BC tumors with high 2HG levels and a distinct DNA methylation pattern associated with worse prognoses. Overall, studies have underscored the critical role of epigenetic-metabolomic interplay in promoting tumorigenesis [159]; in particular, high 2-HG levels induce epigenetic reprogramming associated with progression in different tumors [160,161]. Interestingly, the silencing of *IDH2* and alcohol dehydrogenase iron containing 1 (*ADHFE1*), two enzymes implicated in the mitochondria-associated α-ketoglutarate–dependent production of 2HG [137,162], prompted a marked reduction of endogenous 2HG in BC cells. Furthermore, *ADHFE1* loss resulted in a moderate but significant inhibition of cell cycle kinetics and reduced migration and invasion, suggesting an oncogenic role for *ADHFE1* in BC. In agreement, several studies have associated high *ADHFE1* expression levels with increased synthesis of 2HG and worse patient prognosis in BC [163,164].

In the context of a multi-omics study focused on lipid metabolism, Hilvo et al. conducted gene silencing experiments on seven enzymes involved in phospholipid remodeling and de novo lipid synthesis [60]. The results established that the individual inhibition of multiple lipid metabolism-regulating genes reduced the growth and viability of BC cell lines, which agrees with studies reporting reduced cell migration, invasion, and tumor proliferation in BC [165,166,167] and other tumor types [168,169,170] following the inhibition of specific lipid metabolism-related enzymes. Interestingly, a more recent study described *FASN*, another enzyme involved in de novo lipid metabolism, as a promising therapeutic target for BC treatment [78].

The results of a multi-omics-based study conducted by Kang et al. revealed lipid composition alterations during the EMT in BC [116]. The inhibition of *ELOVL2* increased malignant potential, higher migration rate, and elevated colony formation. Mechanistically, downregulation of *ELOVL2* increased sterol regulatory element-binding transcription factor 1 (*SREBP1*) expression in BC cells and activated lipogenesis, a process associated with the promotion of malignant BC phenotypes. *SREBP1* is a crucial regulator of fatty acid metabolism and plays a pivotal role in the transcriptional regulation of different lipogenic genes mediating lipid synthesis [171,172]. *SREBP1* overexpression has been observed in different tumor types, including BC [60,173,174], and supports the malignant BC phenotype [167]. Finally, Camarda et al. suggested the inhibition of FAO as a novel therapeutic target in a MYC-driven model of TNBC [102]. Based on the characterization of the effects of small-molecule inhibition and knock-down of carnitine palmitoyltransferase 1A (*CPT1*) and carnitine palmitoyltransferase 2 (*CPT2*) in BC cell lines, the authors demonstrated that FAO plays an essential role in this BC model. Furthermore, in vivo experiments demonstrated that treatment with etomoxir, a *CPT1* inhibitor, significantly attenuated tumor growth in various BC models. Additional studies have also demonstrated that individual knockdown of both CPT enzymes reduces FAO metabolism and cell proliferation in different tumors [175,176,177]. In addition, more recent studies suggested *CPT1* as a potential BC tumor target [103,178].

## 5. Future Perspectives and Conclusions

The application of single-omic approaches has significantly contributed to the characterization of BC tumors’ metabolic profile and the identification of BC-specific metabolic biomarkers. Individual omics have utility in clinical practice and can aid early diagnosis and identify indicators of disease progression. Among these experimental approaches, metabolomics represents a non-invasive strategy that could facilitate the development of non-invasive biomarkers with enormous potential for high-risk population screening, patient stratification, and treatment follow-up. However, the metabolome coverage remains limited compared to the genomic or transcriptomic layer, which limits the interpretation of the final results [179]. Moreover, compared with other omics platforms, metabolomics datasets suffer from a lower level of standardization, and we lack extensive data repositories [180,181,182]. Combining different omics datasets and taking advantage of the associated enhancement of statistical power may provide a more powerful strategy to characterize robust and consistent metabolic changes underlying disease states and identify novel therapeutic strategies [183]. Overall, studies describing metabolic alterations in glycolysis, fatty acid, nucleotide, lipid, and amino acid metabolism, could be employed to develop BC biomarkers and identify targets of great interest in clinical practice. This review focused on those multi-omic studies leading to the identification of metabolic markers with clinical utility for the early diagnosis, prognosis, and subtyping of BC tumors and the development of novel therapeutic strategies for BC based on metabolic-associated alterations. Multi-omics approaches have also been used to analyze the potential relationship between particular clinical variables (e.g., obese/non-obese [51]) and higher risk of BC. These studies have paved the way for the application of multi-omic approaches to the exploration of how patient-specific data affect the risk of BC (i.e., diabetes, metabolic syndrome, insulin resistance). Nevertheless, while certain studies included a vast number of samples [59,60,68,69,106], only a few include external independent cohorts of patients to assess the clinical significance of the results. Thus, future studies should analyze larger integrated sample cohorts that could generate sufficiently well-powered datasets and produce accurate and robust findings to validate the clinical utility of these findings. In these studies, coordinated sample processing poses technical challenges, given that each omics platform has specific requirements for sample treatment, and accessibility of available patient material is usually limited [184]. The lack of gold standard unified sample processing workflows and data analysis protocols for normalization, transformation, and scaling to ensure robustness and reproducibility of the results represent challenges to the integration of different datasets [185]. Moreover, in most reviewed studies, analyses of the different omics datasets were conducted individually before the combination of results, reflecting the lack of implementation of specific integration tools directed to the analysis of metabolic alterations. Therefore, developing new computational approaches that could facilitate the integrated analysis of different multi-omics datasets focusing on cancer metabolism would be incredibly beneficial [186]. In this scenario, the application of machine learning approaches to analyze multi-omics datasets could provide novel insights into the characterization of specific BC metabolic phenotypes [187].

## Figures and Tables

**Table 1:** Multi-omics studies focused on the identification of BC metabolic biomarkers.

Study	Sample	Omics Data	Major Findings *
Iqbal et al. [59]	Tissue	M+T	↑ glucose-6-phosphate, 3-phosphoglycerate, and fructose-6-phosphate ↑ *CBX2* and ↓ *CBX7*
Hilvo et al. [60]	Tissue	L+T	↑ *ACACA*, *FASN*, *INSIG1*, and *SREBP1* ↑ phospholipids, sphingomyelins, and ceramides
Luo et al. [61]	Plasma + Tissue	M+T	↓ guanine and hypoxanthine ↓ *PNP* and *HPRT1*
Huang et al. [57]	Plasma + Serum + Tissue	M+T	↑ hypotaurine, glutamate and ↓ oxoglutarate ↑ *GAD1*, *CSAD* and ↓ *GPT*, *GPT2*, *GLUD1*
Dowling et al. [62]	Serum	M+P	↑ glutamate, 12-hydroxyeicosatetraenoic acid, andβ-hydroxybutyrate ↑ coagulation factor V and matrix metalloproteinase 1

*ACACA*: acetyl-CoA carboxylase alpha, BC: breast cancer, *CBX2*: chromobox 2, *CBX7*: chromobox 7, *CSAD*: cysteine sulfinic acid decarboxylase, *GAD1*: glutamate decarboxylase 1, *GLUD1*: glutamate dehydrogenase, *GPT*: glutamic-pyruvic transaminase, *GPT2*: glutamic-pyruvic transaminase 2, *FASN*: fatty acid synthase, *HPRT1*: hypoxanthine phosphoribosyltransferase, *INSIG1*: insulin-induced gene 1, L: lipidomics, M: metabolomics, P: proteomics, *PNP*: purine nucleoside phosphorylase, *SREBP1*: sterol regulatory element-binding transcription factor 1, T: transcriptomics. * Direction of variation, considering the healthy group as a reference.

**Table 2:** Multi-omics studies focused on BC tumor metabolic subtyping.

Study	Sample	Omics Data	Group Comparison	Major Findings *
Moestue et al. [63]	Tissue	M+T	Basal- vs. luminal-like	↑ GPC/PCho and glycine ↓ *CHKA/B* ↑ *PLA2G4A, PLB1, CHDH* and *SARDH*
Grinde et al. [64]	Tissue	M+T	Basal- vs. luminal-like	↓ PCho/GPC
Putluri et al. [65]	Cell lines + Tissue	M+T	Basal- vs. luminal-like	↓ phenylalanine, tryptophan, tyrosine, BCAA, lauric acid and oleic acid ↑ guanine, adenine, thymine, uracil, xanthine, and guanosine
Mahendralingam et al. [66]	Tissue	P+T	Basal- vs. luminal-like	↑ glycolysis (*PFKM, ALDOC, GAPDH,* and *PKM*) and ↓ OXPHOS
Tang et al. [67]	Tissue	G+M+T	ER+ vs. ER-	↓ carnitine derivates and short- and medium-chain fatty acids ↑ long-chain fatty acids and monoacylglycerols
Barupal et al. [68]	Tissue	M+P+T	ER+ vs. ER-	↑ R5P, adenine, guanosine, guanine, xanthine, and hypoxanthine and β-alanine ↑ *G6PD, PGD, TKT, PGM1, RPIA, DERA*
Hilvo et al. [60]	Tissue	L+T	ER+ vs. ER-	↑ palmitate and myristic acid
Haukaas et al. [48]	Tissue	M+T+P	Primary BC subtyping	MC1: ↑ GPC, PCho ↓ acetate and glutamine ↑ *CHKA* ↓ *ALDH* and *GLS* MC2: ↑ glucose MC3: ↑ lactate and alanine
Gong et al. [69]	Tissue	M+T	TNBC subtyping	MPS1: ↑ myristic, palmitoleic, oleic, and arachidonic acid ↑ *ACACA, HMGCR, FASN,* *SCD* MPS2: ↑ glucose 1-phosphate, dihydroxyacetone phosphate, lactate and adenosine 3′ 5′ -diphosphate and ↓ glucose ↑ *PFKP, ENO2, TYMS, CTPS1,* *SLC2A1, SLC16A1*

*ACACA*: acetyl-CoA carboxylase alpha, *ALDH*: aldehyde dehydrogenase, *ALDOC:* aldolase, fructose-bisphosphate C, BC: breast cancer, BCAA: branched-chain amino acid, *CHDH*: choline dehydrogenase, *CHKA*: choline kinase alpha, *CHKB*: choline kinase beta, *CTPS1*: CPT synthase 1, *DERA*: deoxyribose-phosphate aldolase, *ENO2*: enolase 2, ER: estrogen receptor, *FASN*: fatty acid synthase, G: genomics, *GAPDH:* glyceraldehyde-3-phosphate dehydrogenase, *GLS*: glutaminase, *GPC*: glycerophosphocholine, *G6PD*: glucose-6-phosphate dehydrogenase, *HMGCR*: HMG-CoA reductase, L: lipidomics, M: metabolomics, OXPHOS: oxidative phosphorylation, P: proteomics, PCho: phosphocholine, *PKFM*: phosphofructokinase (muscle), *PFKP*: phosphofructokinase (platelet), *PGD*: phosphogluconate dehydrogenase, *PGM1*: phosphoglucomutase 1, *PKM:* pyruvate kinase M1/2, *PLA2G4A*: phospholipase A2 group IVA, *PLB1*: phospholipase B1, PPP: pentose phosphate pathway, *RPIA*: ribose 5-phosphate isomerase 1, R5P: ribose-5-phosphate, *SARDH*: sarcosine dehydrogenase, *SCD*: stearoyl-CoA desaturase, *SLC2A1*: solute carrier family 2 member 1, *SLC16A1*: solute carrier family 16 member 2, T: transcriptomics, TCA: tricarboxylic acid, TKT: transketolase, TNBC: triple negative breast cancer, *TYMS*: thymidylate synthetase. * Direction of variation, considering luminal or ER+ BC group as a reference.

**Table 3:** Multi-omics studies focused on identifying metabolic alterations associated with BC prognosis.

Study	Sample	Omics Data	Major Findings *
Putluri et al. [65]	Cell lines + Tissue	M+T	↑ *RRM2* (pyrimidine metabolism)
Luo et al. [61]	Blood + Tissue	M+T	↑ *RRM2* (pyrimidine metabolism) and ↓ *AMPD1* (de novo purine metabolism)
Iqbal et al. [59]	Tissue	M+T	↑ *CBX2* and ↓ *CBX7* (glycolysis)
Camarda et al. [102]	Cell lines + Tissue	M+T	↓ ACC2 (FAO)
Kang et al. [116]	Cell lines	L+T	↓ *ELOVL2* (lipid synthesis)
Terunuma et al. [107]	Tissue	M+T+P+E	↑ 2HG, SAM and SAH ↑ *IDH2* (glutamine metabolism)
Budczies et al. [106]	Tissue	M+T	↓ *ABAT*, ↑ β-alanine (β-alanine metabolism)

*ABAT*: 4-aminobutyrate aminotransferase, ACC2: acetyl-CoA carboxylase 2, *AMPD1*: adenosine monophosphate deaminase 1, *CBX2*: chromobox 2, *CBX7*: chromobox 7, E: epigenomics, *ELOVL2*: ELOVL fatty acid elongase 2, FAO: fatty acid oxidation, L: lipidomics, *IDH2*: isocitrate dehydrogenase (NADP(+)) 2, M: metabolomics, P: proteomics, *RRM2*: ribonucleotide reductase regulatory subunit M2, SAH: S-adenosylhomocysteine, SAM: S-adenosyl- methionine, T: transcriptomics, 2HG: 2-hydroxyglutarate. * Direction of metabolic alterations directly correlated with worse BC patients’ outcomes.

**Table 4:** Multi-omics studies focused on developing new therapeutic strategies for the treatment of BC.

Study	Omics Data	BC Subtype	Potential Targets
Iqbal et al. [59]	M+T	TNBC and luminal-like	*CBX2* and *CBX7*
Gong et al. [69]	M+T	TNBC	*FASN* and *LDH*
Mahendralingam et al. [66]	P+T	Basal- and luminal-like	*GLUT1*, *HK*, *LDH,* and *PDC*
Putluri et al. [65]	M+T	Basal- and luminal-like	*RRM2*
Terunuma et al. [107]	M+T+P+E	TNBC and basal-like	*ADHFE1*
Hilvo et al. [60]	L+T	TNBC, luminal- and basal-like	*ACACA, ELOVL1, FASN, INSIG1, SCAP, SCD* and *THRSP*
Kang et al. [116]	L+T	Luminal-like	*ELOVL2*
Camarda et al. [102]	M+T	TNBC and HER2 +	*CPT1* and *CPT2*

*ACACA*: acetyl-CoA carboxylase alpha, *ADHFE1*: alcohol dehydrogenase iron containing 1, *CBX2*: chromobox 2, *CBX7*: chromobox 7, *CPT1*: carnitine palmitoyltransferase 1A, *CPT2*: carnitine palmitoyltransferase 2, E: epigenomics, *ELOVL1*: ELOVL fatty acid elongase 1, *ELOVL2*: ELOVL fatty acid elongase 2, *FASN*: fatty acid synthase, *GLUT1*: glucose transporter 1, *HK*: hexokinase, *IDH2*: isocitrate dehydrogenase (NADP(+)) 2, *INSIG1*: insulin-induced gene 1, L: lipidomics, *LDH*: lactate dehydrogenase, M: metabolomics, P: proteomics, *PDC*: pyruvate dehydrogenase kinase, *RRM2*: ribonucleotide reductase regulatory subunit M2, *SCAP*: SREBF chaperone, *SCD*: stearoyl-CoA desaturase, T: transcriptomics, TNBC: triple negative breast cancer, *THRSP*: thyroid hormone-responsive.
